# Venezuelan Equine Encephalitis Virus nsP3 Phosphorylation Can Be Mediated by IKKβ Kinase Activity and Abrogation of Phosphorylation Inhibits Negative-Strand Synthesis

**DOI:** 10.3390/v12091021

**Published:** 2020-09-13

**Authors:** Allison Bakovic, Nishank Bhalla, Stephanie Kortchak, Chengqun Sun, Weidong Zhou, Aslaa Ahmed, Kenneth Risner, William B. Klimstra, Aarthi Narayanan

**Affiliations:** 1 National Center for Biodefense and Infectious Diseases, George Mason University, Manassas, VA 20110, USA; abakovic@masonlive.gmu.edu (A.B.); nbhalla@gmu.edu (N.B.); skortcha@masonlive.gmu.edu (S.K.); aahmed30@masonlive.gmu.edu (A.A.); krisner@masonlive.gmu.edu (K.R.); 2 Center for Vaccine Research, Department of Immunology, University of Pittsburgh, Pittsburgh, PA 15261, USA; chs79@pitt.edu (C.S.); klimstra@pitt.edu (W.B.K.); 3 School of Systems Biology, George Mason University, Manassas, VA 20110, USA; wzhou@gmu.edu

**Keywords:** Venezuelan equine encephalitis virus, phosphorylation, IKK complex, IKKβ kinase assay, replication-deficient, non-structural protein 3, mutations, revertants, negative-strand, sequencing, phosphomimetics

## Abstract

Venezuelan equine encephalitis virus (VEEV), a mosquito transmitted alphavirus of the *Togaviridae* family, can cause a highly inflammatory and encephalitic disease upon infection. Although a category B select agent, no FDA-approved vaccines or therapeutics against VEEV currently exist. We previously demonstrated NF-κB activation and macromolecular reorganization of the IKK complex upon VEEV infection in vitro, with IKKβ inhibition reducing viral replication. Mass spectrometry and confocal microscopy revealed an interaction between IKKβ and VEEV non-structural protein 3 (nsP3). Here, using western blotting, a cell-free kinase activity assay, and mass spectrometry, we demonstrate that IKKβ kinase activity can directly phosphorylate VEEV nsP3 at sites 204/5, 142, and 134/5. Alanine substitution mutations at sites 204/5, 142, or 134/5 reduced VEEV replication by >30-100,000-fold corresponding to a severe decrease in negative-strand synthesis. Serial passaging rescued viral replication and negative-strand synthesis, and sequencing of revertant viruses revealed reversion to the wild-type TC-83 phosphorylation capable amino acid sequences at nsP3 sites 204/5, 142, and 135. Generation of phosphomimetic mutants using aspartic acid substitutions at site 204/5 resulted in rescue of both viral replication and negative-strand RNA production, whereas phosphomimetic mutant 134/5 rescued viral replication but failed to restore negative-strand RNA levels, and phosphomimetic mutant 142 did not rescue VEEV replication. Together, these data demonstrate that IKKβ can phosphorylate VEEV nsP3 at sites 204/5, 142, and 134/5, and suggest that phosphorylation is essential for negative-strand RNA synthesis at site 204/5, but may be important for infectious particle production at site 134/5.

## 1. Introduction

Venezuelan equine encephalitis virus (VEEV), a New World alphavirus, is a member of the *Togaviridae* family of RNA viruses. Infection with VEEV can progress to encephalitic disease that is fatal in ~1% of cases [1,2,3] whereas Old World alphavirus infection (e.g., Sindbis, Chikungunya and Semliki Forest viruses) is primarily associated with arthritogenic disease outcomes [4]. Although VEEV is endemic to South America, its spread to the southern United States has caused periodic outbreaks affecting both equine and human populations, with an estimated 21 outbreaks in the Americas since the 1930s [5]. VEEV is an arbovirus transmitted naturally by mosquitoes, however aerosol infection is also possible as demonstrated by past occurrences of laboratory-acquired infections [6] and VEEV has previously been developed as a bioweapon [7]. Despite being classified as a category B select agent pathogen by the National Institute of Allergy and Infectious Diseases (NIAID), no FDA-approved vaccines or therapeutics against VEEV exist [2,8].

The VEEV genome consists of a positive-sense, single stranded RNA molecule ~11.4 kb in length that encodes two polyproteins [1,5]. The non-structural polyprotein consists of the four non-structural proteins nsP1-4, that drive viral RNA replication in concert with a range of host proteins [9]. The structural polyprotein is cleaved into the viral capsid and glycoproteins, which are responsible for packaging genomes into progeny virions [1,10]. While multiple activities of nsP1, 2, and 4 have been described, the activities of nsP3 have only recently begun to be elucidated [9]. Three distinct domains in nsP3 have been categorized; an N-terminal macrodomain conserved among many alphaviruses, which exhibits homology with other RNA viruses, an alphavirus unique domain (AUD), and a C-terminal hypervariable domain (HVD) [11,12]. The macrodomain binds ADP-ribose and possesses phosphatase and de-ADP-ribosylation activity [12,13]. The AUD encompasses a zinc-ion coordination site and amino acid site mutations in this domain in Sindbis virus (SINV) have revealed conserved, individual cysteine residues that are vital for viral replication [10]. The HVD contributes to cell-specific preferences for host factor interactions between different alphaviruses [14]. Hyperphosphorylation of several HVD threonine and serine residues is critical for viral RNA synthesis in both New World and Old World alphaviruses [14,15,16,17,18]. Recent work has begun to elucidate the structural and functional characteristics of VEEV nsP3; however, the interplay between nsP3 and host factors is not well understood.

The nuclear factor-κB (NF-κB) signaling pathway regulates the transcription of genes associated with cellular stress, apoptosis, proliferation, and immune responses [19,20,21]. The NF-κB complex is normally inactive, bound by inhibitor of κB (IκB) proteins and sequestered in the cytoplasm of eukaryotic cells [22]. Upon stimulation, the inhibitor of κB kinase (IKK) complex is activated and phosphorylates IκB proteins, which are subsequently ubiquitinated and degraded [22], unmasking nuclear localization signals in the p65/p50 subunits of NF-κB to allow nuclear translocation and induction of various target genes [23]. The IKK complex, comprised of IKKα, IKKβ, and IKKγ is the master regulator of this signaling cascade and plays an important pro-inflammatory role during most viral infections [24,25]. Many viruses disrupt the NF-κB signaling cascade either by encoding proteins to evade the pro-inflammatory responses upregulated by the pathway or by directly hijacking the pathway through modulation of IKK complex activity [26,27,28,29]. For example, Influenza virus and adenovirus trigger NF-κB activation through accumulation of viral proteins in the endoplasmic reticulum and subsequent activation of the IKKβ subunit which is a necessary intermediate for cascade activation [30]. Similarly, the human T-lymphotropic virus Tax oncoprotein directly interacts with the IKKγ subunit by recruiting kinase MEKK1 for phosphorylation of the IKK complex [28]. In these two examples, the IKK complex is targeted to control induction of apoptotic regulatory genes triggered by NF-κB activation and promote transcription of cellular growth genes in order to promote viral replication [30]. Thus, targeted inhibition of NF-κB pathway members, specifically the IKK complex, could exert antiviral effects. For example, cyclopentenone prostanoids directly bind to IKKβ, and treatment has shown reduction in IKK activity, infectious virion production, and viral gene expression in in vitro models following infection with herpes simplex virus-1 [30]. Collectively, these examples suggest IKK complex and NF-κB are appealing targets for novel antiviral therapeutics.

Previous research has demonstrated NF-κB cascade activation and macromolecular reorganization of the IKK complex, in particular IKKβ, during infection of U-87MG astrocyte cells with the live-attenuated TC-83 vaccine strain of VEEV [31]. Treatment with the IKKβ inhibitor BAY-11-7082 significantly decreased levels of both infectious virion particles and viral RNA copies in vitro and in vivo [31], whereas overexpression of IKKβ increased viral titers following infection. Furthermore, infection of IKKβ^−/−^ cell lines demonstrated a decrease in viral replication, suggesting that IKKβ activity is important for VEEV replication. Additionally, a direct interaction between VEEV nsP3 and IKKβ was confirmed by both mass spectrometry and confocal microscopy [31]. Inhibition of IKKβ kinase activity decreased viral replication but had no effect on downstream NF-κB activation of transcription [31], suggesting that VEEV replication is enhanced by an activity of IKKβ, possibly mediated by phosphorylation of one or more viral proteins, potentially nsP3.

In this study, we investigated IKKβ mediated phosphorylation of nsP3 and evaluated the effects of nsP3 phosphorylation on VEEV replication. NsP3 phosphorylation levels in cells were diminished upon treatment with a small molecule inhibitor of IKKβ, BAY-11-7082, as measured by western blotting. A cell-free, in vitro kinase activity assay performed with IKKβ and purified VEEV nsP3 in the presence of ^33^P-γ-ATP demonstrated direct phosphorylation of VEEV nsP3 by IKKβ, with the observed phosphorylation signal significantly higher than that of autophosphorylation or a negative control protein, GAPDH. Using mass spectrometry, we identified 18 amino acids in nsP3 that exhibited phosphorylation following VEEV TC-83 infection of U-87MG astrocytes and mouse embryonic fibroblast (MEF) cells competent for IKKβ activity. Inhibition of IKKβ activity using BAY-11-7082 in U-87MG astrocytes resulted in abrogation of phosphorylation of eleven amino acids, whereas infection of IKKβ^−/−^ MEF cells resulted in abrogation of phosphorylation of four amino acids. Charge-to-alanine substitutions were created at 12 phosphorylation sites of interest to further evaluate the effect of nsP3 phosphorylation on viral replication. We identified two nsP3 sites, 204/5 and 142, where alanine substitution abrogated production of detectable infectious particles when introduced in both the TC-83 vaccine strain and the Trinidad Donkey (TrD) epizootic wild-type strain of VEEV. Additionally, alanine substitution at site 134/5 displayed an intermediate phenotype with replication ~10^5^-fold lower when compared to replication of TC-83 and ~30-fold lower when compared to replication of TrD. Furthermore, these mutants were unable to form detectable infectious particles following C6/36 mosquito cell infection. Analysis of positive- and negative-strand RNA levels indicated that phosphorylation-deficient mutants synthesized significantly lower levels of negative-strand during infection when compared to TC-83. Results from a cell-free, in vitro kinase activity assay displayed a reduction in phosphorylation of purified nsP3 mutant proteins with alanine substitutions at sites 204/5, 142 or 134/5 by IKKβ in comparison to wild-type nsP3. Serial passaging of the replication-deficient mutants generated revertants with a replication competent phenotype, attributed to a reversion of the alanine substitutions back to the original phosphorylation-capable TC-83 amino acids. Furthermore, phosphomimetic mutants with aspartic acid substitutions at nsP3 sites 204/5, 142, and 134/5 were generated and tested for replication competence. Phosphomimetic mutant 204/5 restored both infectious particle production and negative-strand RNA synthesis almost comparable to levels observed in TC-83 infected cells, whereas phosphomimetic mutant 134/5 partially restored the ability to produce infectious particles but did not increase negative-strand RNA synthesis. The phosphomimetic mutant 142 did not rescue viral replication. Treatment of phosphomimetic mutants with the IKKβ inhibitor BAY-11-7082 had little effect on levels of infectious particle production. Overall, we identify IKKβ as the first reported host kinase capable of phosphorylating VEEV nsP3 on residues 204/5, 142, and 134/5 and demonstrate that phosphorylation of residues 204/5 is essential for negative-strand synthesis while phosphorylation of residues 134/5 may be important for infectious particle production.

## 2. Materials and Methods

### 2.1. Cell Culture

Vero African green monkey kidney cells (ATCC, CCL-81), U-87MG human astrocytoma cells (ATCC, HTB-14), C6/36 *Aedes albopictus* mosquito cells (ATCC, CRL-1660), and BHK-21 baby hamster kidney cells (ATCC, CCL-10) were obtained from the American Type Culture Collection (Manassas, VA, USA). Inhibitory κB kinase KO (IKKβ^−/−^) and WT MEF cells were a kind gift from Dr. Cynthia Masison from NIH/NCI [32,33]. Knockout of IKKβ expression in these cells was previously validated [31]. U-87MG, Vero, BHK-21, and MEF cells were cultured in Dulbecco’s Modified Eagle’s Medium (DMEM, 112-013-101CS, Quality Biological, Gaithersburg, MD, USA) supplemented with 4.5 g/L glucose, 2 mM L-glutamine (MT2005CI, FisherSci, Chicago, IL, USA,), 10% heat-inactivated fetal bovine serum (10437028, ThermoFisher, Carlsbad, CA, USA) for U-87MG and MEF cells or 5% heat-inactivated fetal bovine essence (10805-184, VWR, Dixon, CA, USA) for Vero and BHK-21 cells with 10 ug/mL streptomycin and 10 U/mL penicillin (45000-652, VWR). C6/36 cells were cultured in Eagle’s Minimum Essential Medium (EMEM, 670086, VWR) supplemented with 10% heat-inactivated fetal bovine serum, 10 ug/mL streptomycin and 10 U/mL penicillin. All cell lines were cultivated at 37 °C and 5% CO_2_.

### 2.2. Expression and Purification of VEEV nsP3

Bacterial expression plasmids encoding VEEV TC-83 nsP3 wild-type (NCBI reference sequence L01443) or mutant nsP3 sequences was constructed using the pET-28a(+) vector containing an N-terminal 6X His-tag sequence and an internal T7-tag sequence with a thrombin cleavage site lac repressor and operator by Twist Biosciences (San Francisco, CA, USA). Expression and purification of nsP3 using *E. coli* was performed by Reaction Biology Corp. (Malvern, PA, USA).

### 2.3. Kinase Profiling Assay

Kinase HotSpot™ Assay for nsP3 substrate vs. IKKβ (Cat #: IKKβ/IKBKB) was performed by Reaction Biology Corp. Briefly, purified VEEV nsP3 was diluted to a concentration of 2 μM in base reaction buffer in duplicate (20 mM Hepes (pH 7.5), 10 mM MgCl_2_, 1 mM EGTA, 0.02% Brij35, 0.02 mg/mL BSA, 0.1 mM Na_3_VO_4_, 2 mM DTT, 1% DMSO). Purified IKKβ kinase was subsequently delivered into the substrate solution at a dosage of 200 nM and mixed gently. The compound mixture was then delivered into a kinase reaction mixture using nanoliter range acoustic technology (Echo550, Reaction Biology Corp., Malvern, PA, USA) and incubated for 20 min at room temperature. ^33^P-γ-ATP, with a specificity of 10 mCi/mL, was delivered into the reaction mixture at a concentration of 10 μM which was subsequently incubated for 1 h at room temperature. Reactions were spotted on P81 ion exchange paper and kinase activity was subsequently detected by the filter-binding method after unreacted phosphate was removed by washing. High throughput screening using a scintillation counter quantitated kinase activity. A negative control reaction contained the IKKβ kinase alone in the absence of the nsP3 substrate (auto-phosphorylation). An inert negative control protein, GAPDH (ab82633, Abcam, Cambridge, MA) diluted to 2 μM in base reaction buffer, was also evaluated with IKKβ in the assay for evaluation of non-specific effects of the kinase. Reaction Biology Corp. positive control for IKKβ activity was a small peptide, IKKtide, used as substrate at 20 μM vs. IKKβ for efficiency evaluation. Graphs display the raw values of ^33^P-γ-ATP counts detected by the scintillation counter and corrected for purity of the input nsP3 substrate proteins. 

### 2.4. Viruses and Plasmids

A plasmid encoding the full-length VEEV vaccine strain TC-83 genome was modified to express nano Luciferase as a cleavable component of the structural polyprotein (TaV-nLuc) as previously described [34,35], and additional nsP3 mutations were generated in this backbone using PCR mutagenesis (Quick Change Kit, Invitrogen, Carlsbad, CA, USA). A pCAGGS-backbone plasmid expressing N-terminally HA-tagged nsP3 of VEEV ZPC738 was previously described [36]. VEEV TC-83 virus not expressing nano Luciferase was obtained from BEI Resources [37].

### 2.5. In Vitro Transcription

Plasmids encoding nsP3 mutant viruses were linearized using MluI digestion and purified using a Minelute PCR Purification Kit (28006, Qiagen, Louisville, KY, USA,). RNA was transcribed from linearized DNA using an Invitrogen Sp6 MegaScript Kit (AM1330, FisherSci,) per manufacturer’s instructions. RNA was purified using a Qiagen RNeasy Kit (74104) and quantitated using a NanoDrop spectrophotometer (ThermoFisher, Waltham, MA, USA).

### 2.6. Electroporation and Viral Cultivation

BHK-21 or Vero cells were seeded and grown to 90% confluency in T-75 flasks. Electroporation with 1µg of in vitro transcribed RNA was performed using BHK-21 cells for TC-83 and Vero cells for TrD at 860 V, 25 µF, 950 Ω in BTX 0.2 cm gap cuvettes (USA Scientific, Ocala, FL, USA, 9104-5050) using an ECM 630 Electroporation System (BTX). Electroporated cells were added to a T-75 flask and supernatants collected at 6, 18, and 24 h post electroporation to harvest viral stocks. Supernatants were subjected to 0.2-micron filtering (09-719-006, FisherSci) and frozen at −80 °C.

### 2.7. Viral Infections and IKKβ Inhibitor Treatment

For all infections, the appropriate cell line was seeded in 96-well plates 24 h prior as follows: U-87MG’s at 1 × 10^4^ and Vero at 5 × 10^4^ per well. C6/36 cells were seeded in 24-well plates 24 h prior at 1 × 10^5^ per well. Vero cells were seeded in 6-well plates 24 h prior at 5 × 10^5^ per well. Viruses were either undiluted, diluted 1:1 in the appropriate culture media, or diluted to the indicated multiplicity of infection (MOI). After a 1-h infection, virus was removed and the appropriate cell media was added. Plates were incubated at 37 °C, 5% CO_2_ for indicated durations. For inhibitor treatment, U-87MG and MEF cells were seeded at a density of 3 × 10^5^ cells per well in 6-well plates. The IKKβ inhibitor BAY-11-7082 (S2913, SelleckChem, Houston, TX, USA) was dissolved in DMSO, diluted in media for a final concentration of 1 µM for U-87MG and MEF cells or 10 µM for Vero cells and cells were pre-treated for 2 h prior to infection. U-87MG and MEF cells were infected with VEEV TC-83 for 1 h at indicated MOI. Vero cells were infected with viruses as described above for 1 h. Conditioned media containing the inhibitor or standard media was added to cells after removal of virus. Plates were incubated at 37 °C, 5% CO_2_ for indicated durations.

### 2.8. Plaque Assay

Vero cells were plated in 12-well plates at a density of 2 × 10^5^ cells/well for 24 h. Viral stocks or infection supernatants were serially diluted to 10^−8^ in culture media and overlaid on cells for 1 h. Cells were covered with Eagle’s Minimum Essential Medium (without phenol red, supplemented with 5% fetal bovine essence, non-essential amino acids, 1 mM sodium pyruvate (45000-710, VWR), 2 mM L-glutamine, 20 U/mL penicillin, and 20 µg/mL streptomycin) containing 0.6% agarose. 48 h post-infection, cells were fixed with 10% formaldehyde (F79P-4, FisherSci) for 1 h. Medium was removed, wells were washed with diH_2_O, and cells were stained with a 1% crystal violet (FisherSci, C581-25) in a 20% ethanol solution (BP2818-4, FisherSci). For plaque assay of VEEV TrD nsP3 mutants, virus was serially diluted in virus diluent (phosphate-buffered saline, 1% donor calf serum) and overlaid on BHK-J cells seeded at a density of 1 × 10^6^ cells/well in 6-well plates for 24 h. Plates were stained with neutral red and plaques were counted. All viral entities were titered in triplicate unless otherwise specified.

### 2.9. Luciferase and Bradford Protein Assay

At indicated time points, supernatants were collected or cellular lysates were obtained using 1X Passive Lysis Buffer (E1941, Promega, Madison, WI, USA) and the Nano-Glo Luciferase Assay System (N1130, Promega) was used to measure luciferase activity per the manufacturer’s instructions. An aliquot of cellular lysates were mixed with Bradford Reagent (5000006, Bio-Rad, Hercules, CA, USA) per the manufacturer’s instructions. A standard curve for total protein was established using Bovine Serum Albumin (BSA, BP1600, FisherSci) diluted in Passive Lysis Buffer at concentrations of 1, 2.5, 5, 10, 15, 20, 25 µg/µL. Mock-infected cells were used to establish the limit of detection for luciferase assays. Luminescence and absorbance was measured using a DTX 880 multimode plate reader (Beckman Coulter, Brea, CA, USA). Intracellular luciferase was normalized to total µg of protein. Overall, sensitivity of the DTX 880 multimode plate reader was somewhat limited due to the age and performance of the machine with lower than anticipated RLU values observed following infection.

### 2.10. RNA Extraction

At indicated time points, cells were washed with phosphate buffered saline (PBS, L0119-0500, VWR) and lysed with TRIzol Reagent (Invitrogen). Intracellular RNA was extracted using MagBead Direct-zol RNA kit or Direct-zol Miniprep RNA kit (Zymo Research, Irvine, CA, USA) per the manufacturer’s instructions. Extracted viral RNA was stored at −80 °C prior to use.

### 2.11. Primer/Probes and cDNA

All primers and probes for detection of viral RNA were designed and obtained from Integrated DNA Technologies (IDT) utilizing the PrimerQuest Tool. Primer/probe sets contained a double-quenched ZEN/IBFQ probe with a 6-FAM fluorescent dye attachment at the 5′ end. Capsid primer/probe set, initially described by Julander et al. [38] was used and is represented in Table 1. Quantitative data for nsP3 is represented for each non-structural RT-PCR figure and the primer/probe set is represented in Table 1. 18S rRNA endogenous control primer/probe set was obtained from ThermoFisher (4333760T). cDNA specific to negative-strand RNA for VEEV TC-83 was generated using a forward primer containing a T7 promoter sequence attached at the 5′ end and a high-capacity cDNA reverse transcription kit (4368814, ThermoFisher) per the manufacturer’s instructions. For qPCR of negative-strand viral RNA, forward primer specific to the T7 promoter sequence and reverse primer specific to VEEV TC-83 were utilized. All primer sets for negative-strand viral RNA quantitation are represented in Table 1.

### 2.12. Semi-Quantitative RT-PCR

Semi-quantitative RT-PCR for targets of capsid, nsP3, and 18S used thermal cycling conditions adapted from Verso 1-step RT-qPCR kit (AB4101C, ThermoFisher) per the manufacturer’s instructions: 1 cycle at 50 °C for 20 min, 1 cycle at 95 °C for 15 min, 40 cycles at 95 °C for 15 s with 51 °C (capsid), 59 °C (nsP3), 60 °C (18S) for 1 min using StepOnePlus™ Real Time PCR system. qRT-PCR for detection of viral negative-strand used thermal cycling conditions adapted from PowerUp SYBR Green (A25742, ThermoScientific) per the manufacturer’s instructions: 1 cycle at 50 °C for 2 min, 1 cycle at 95 °C for 2 min, 40 cycles at 95 °C for 15 s, 60 °C for 15 s and 72 °C for 1 min using StepOnePlus™ Real Time PCR system. A no template cDNA control and mock infections were included for all analyses and established the limits of detection. Semi-quantitative RT-PCR values were calculated using the ΔΔCt method [39] with viral entities normalized to 18S levels.

### 2.13. Transfections

U-87MG cells were seeded at a density of 3 × 10^5^ cells per well in 6-well plates. Transfections used Attractene Transfection Reagent (301005, Qiagen) with 1 µg of DNA plasmid transfected per well as per manufacturer’s instructions. The IKKβ inhibitor, BAY-11-7082, was diluted to 1 µM with culture media and added to the appropriate treatment well at the time of transfection. A pcDNA3.1 (+) plasmid was included as a negative control. Plates were incubated at 37 °C, 5% CO_2_ for 24 h.

### 2.14. Western Blot

For preparation of whole cell lysates from infected or transfected cells, media was removed and cells washed twice with PBS. Cells were lysed with clear lysis buffer (CLB) supplemented with 10 mM 1,4-dithiothreitol (DTT, P2325, Invitrogen). CLB contained 50 mM Tris-HCL at pH 7.5 (351-006-101, Quality Biological), 120 mM NaCl (S271-500, FisherSci), 5 mM EDTA (351-027-721, Quality Biological), 0.5% NP-40 (492016, EMD Millipore, Burlington, MA, USA), 50 mM NaF (S7920, Sigma-Aldrich, Milwaukee, WI, USA), 0.2 mM Na_3_VO_4_ (450243, Sigma-Aldrich), protease inhibitor tablet (88666, ThermoFisher) and phosphatase inhibitor cocktail (78420, ThermoFisher). Protein lysates were vortexed every 5 min for 20 min and supernatants were collected after centrifugation at 10,000 rpm for 10 min at 4 °C. Sample supernatants were diluted 1:1 in 2× Laemmli buffer (1610737, Bio-Rad) supplemented with DTT and boiled for 10 min. Lysates were separated on 4–20% Tris-Glycine Gels and transferred to polyvinyl difluoride (PVDF) membranes via wet transfer for 2 h at 250 mA. Membranes were blocked at room temperature for 30 min using 5% BSA in Tris-buffered saline with 0.1% Tween-20 (TBS-T). Anti-phospho serine antibody (ab9332, Abcam), anti-phosphothreonine antibody (ab9337, Abcam), anti-phospho tyrosine antibody (ab17302, Abcam, 61-5800, Invitrogen) or anti-HA tag antibody (ab18181, Abcam) were diluted in 5% BSA in TBS-T at 4 µg/mL, 2 µg/mL, 1:1000, and 1:1000 respectively, and incubated on individual membranes overnight at 4 °C. Following 3 5-min washes with TBS-T, membranes were incubated with respective secondary HRP-conjugated antibodies (PI32460, FisherSci) diluted 1:10,000 in 5% BSA in TBS-T at room temperature for 1 h and then washed twice with TBS-T and twice with TBS. Membranes were imaged using SuperSignal West Femto Maximum Sensitivity Substrate Kit (34095, ThermoFisher) and a Bio-Rad Molecular Imager ChemiDoc XRS system. Phosphorylation signals were calculated using NIH ImageJ software and values were normalized to both the actin loading control and the HA-tag for all lanes and quantitation is presented as percent fold-change vs. wild-type.

### 2.15. Liquid Chromatography-Mass Spectrometry

LC-MS/MS was performed as previously described [40]. Briefly, VEEV TC-83 infected U-87MG or MEF cells were trypsinized (25200056, ThermoFisher) and washed twice with PBS using a centrifugal spin of 1500 rpm for 5 min during each wash. Cellular pellets were lysed using 8 M urea, disulfide bonds were reduced with 1 M DTT and alkylated with iodacetamide. Trypsin digestion was performed for 4 h at 37 °C and peptides were eluted with ZipTip purification (Z720070, Millipore,). LTQ-tandem MS/MS with nanospray reverse-phase liquid chromatography (ThermoFisher) was performed. Post sample injection, column washes proceeded for 5 min with 0.1% formic acid at 200 nL/min. Peptides were then eluted on a 50-min linear gradient from 0–40% acetonitrile in 0.1% formic acid followed by a 5-min holding step in 80% acetonitrile in 0.1% formic acid. LTQ-MS operated in data-dependent mode and each full MS scan was followed with five MS-MS scans where the five most abundant molecular ions were dynamically selected and fragmented by collision-induced dissociation using normalized collision energy of 35%. Mass spectra were fitted against NCBI reference sequence NP_740698.1 or L01443 for putative VEEV nsP3 post translational modification analysis with Sequest Bioworks software (ThermoFisher).

### 2.16. Serial Passaging of Virus

Vero cells were seeded in 6-well plates at varying densities of 5 × 105, 2.5 × 105, and 1.5 × 105 per well to ensure 90% confluency prior to infection. On the first day of infection, cells were infected in duplicate with 250 µL of undiluted virus. Following a 1 h infection, 250 µL of fresh medium (FM) was added. Plates were incubated for 24 h. The following day, 250 µL of supernatants from the initial plate were added to fresh 6-well plates containing 250 µL of FM. The newly infected plates were incubated for 24 h and this was continued for 20 passages. At passages 5, 10, 15, and 20, all media was removed, wells washed with PBS, and cells lysed with TRIzol reagent. Intracellular RNA was extracted as previously described. For each passage, the remaining viral supernatants were collected and evaluated for infectious titers via plaque assay.

### 2.17. Sequencing

cDNA for the intracellular RNA extracted at serial passages 5, 10, 15, 20 was generated using a high-capacity cDNA reverse transcription kit (4368814, ThermoFisher) and a T7-tagged reverse primer targeted to the 3′ end of nsP3 or the 3′UTR containing the CSE sequence on the positive-strand of VEEV TC-83. cDNA was amplified using a T7 primer and a forward primer targeting the 5′ end of the nsP3 and CSE sequences of VEEV TC-83 using PlatinumSuperFi PCR master mix kit (12358-050, Invitrogen): 1 cycle at 98 °C for 30 s, 35 cycles at 98 °C for 5 s, 51 °C for 10 s, 72 °C for 4 min, and 1 cycle at 72 °C for 5 min. All primer sets are represented in Table 2. Forward primers were designed every 800 base-pairs along the nsP3 and 3′UTR segment of the VEEV TC-83 genome and used to sequence viral cDNA, performed using Sanger Sequencing by Macrogen Corp. (Rockville, MD, USA). SnapGene Viewer was utilized to view chromatograms.

### 2.18. Bioinformatics

Phosphorylation site predictions on VEEV nsP3 were performed with the VEEV TC-83 sequence (L01443) and the NetPhos 3.1 Server with results filtered for hits scored above 0.5. All sequence alignments utilized Clustal Omega (EMBL-EBI). DNA to protein analysis was performed with ExPASy SIB Bioinformatics Resource Portal. The following sequences obtained from the NCBI nucleotide database were used for alignments: VEEV Enzootic strain NP_740698.1, VEEV TrD strain L01442, VEEV TC-83 strain L01443, EEEV FL93 Strain ABL84686.1, SINV TR339 Strain (William Klimstra), CHIKV LR Strain AQX78116.1.

### 2.19. Imaging

Undiluted viral supernatants from serial passages 11–13 for mutants 204/5 and 142 and passage 5 for mutant 134/5 were used to infect Vero cells in a 96-well plate for 24 h. Wild-type VEEV TC-83 and mock infection controls were included. At 24 hpi, media was removed, cells washed with PBS, and fixed with 4% paraformaldehyde. Cells were imaged using EVOS™ FL Auto Imaging System (ThermoFisher). Images were taken using 4X objective with brightness adjusted to 50% and photo viewer clarity set to 100. Inset images were taken using 20× objective with brightness adjusted to 70% and photo viewer clarity set to 100. Scale bars for 4× and 20× images are 1000 µm and 200 µm, respectively.

### 2.20. Statistical Analyses

All graphs represent the mean ± SD for all data obtained. Prism 7 (Graph Pad, San Diego, CA, USA) was used for all statistical analyses and statistical significance was determined using Two-Way ANOVA with Dunnett’s Post Test unless otherwise stated. Significance values are indicated using asterisks for * *p* < 0.0332, ** *p* < 0.0021, *** *p* < 0.0002, and **** *p* < 0.0001.

## 3. Results

### 3.1. Disruption of IKKβ Activity Reduces nsP3 Phosphorylation and Viral Replication

**Figure 1:**
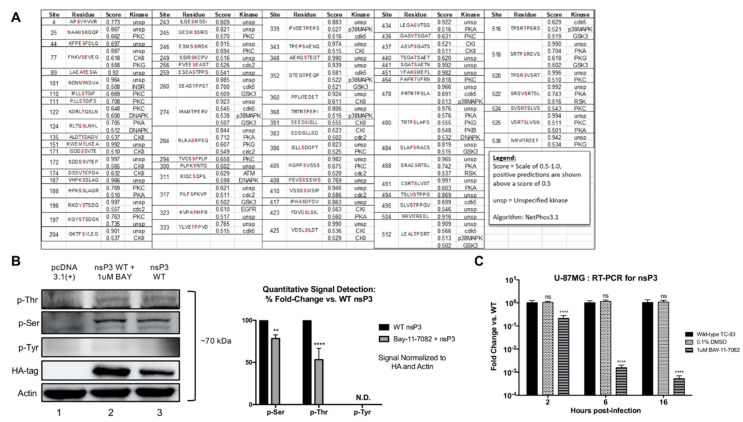
VEEV nsP3 is phosphorylated and inhibition of IKKβ reduces nsP3 phosphorylation and viral replication levels. (**A**) VEEV nsP3 amino acid residues predicted to be phosphorylated by host kinases were identified using the NetPhos 3.1 server and VEEV TC-83 L01443 sequence obtained from NCBI. (**B**) pcDNA 3.1(+) empty vector or pCAGGS plasmid expressing N-terminus HA-tagged wild-type VEEV ZPC738-nsP3 were transfected in U-87MG cells in the presence or absence of the IKKβ inhibitor BAY-11-7082 (1 µM) for 24 h. Lysates were probed for phospho-serine, phospho-threonine, phospho-tyrosine, or HA-tag and signals were calculated and normalized as described in Section 2. Western blot and graphical data are representative of three independent experiments (*n* = 3). ND represents undetectable virus (**C**) U-87MG cells were treated with DMSO or the IKKβ inhibitor BAY-11-7082 (1 µM) for 2 h, subsequently infected with TC-83 at MOI of 0.1 in triplicate for 1 h and conditioned media containing BAY-11-7082 or standard media was replaced after removal of virus. At 2, 6, 16 hpi, RNA was collected, and RT-PCR performed as described in Section 2. Graph represents data from two independent experiments performed in triplicates (*n* = 6). ** *p* < 0.0021 and **** *p* < 0.0001, ns, not significant.

The alphavirus nsP3 is hyperphosphorylated on multiple amino acid residues during infection, which may be important for regulation of RNA synthesis [12,41,42]. However, the cellular kinase or kinases mediating phosphorylation of nsP3 of both New World and Old World alphaviruses have yet to be identified. We identified potential sites phosphorylated on VEEV nsP3 in silico (Figure 1A) using the Reference Sequence L01443 for VEEV TC-83 and the prediction software, NetPhos3.1. Sites were identified with a confidence threshold set at 0.5, indicating a positive prediction. 73 sites on nsP3 were predicted to be phosphorylated by a variety of kinases. As we previously demonstrated an interaction between IKKβ and nsP3 [31], we first confirmed nsP3 phosphorylation using U-87MG astrocytes, as these cells are susceptible to VEEV infection in vitro [43]. Cells were transfected with an empty control vector or a plasmid expressing HA-tagged nsP3 in the presence or absence of the IKKβ inhibitor, BAY-11-7082. Cellular lysates were probed for the presence of phospho-serines, phospho-threonines, and phospho-tyrosines. Our results demonstrated the presence of phosphorylation of nsP3 on one or more serine or threonine residues (Figure 1B). Further, the IKKβ inhibitor treated cells displayed an ~18% reduction of phosphorylation on nsP3 serines (*p <* 0.0021) and a ~34% reduction of phosphorylation on nsP3 threonines (*p <* 0.0001) when compared to the untreated cells expressing nsP3. A greater amount of nsP3 was present in BAY-11-7082 treated cells as BAY-11-7082 can also inhibit proteasome activity [44]. Phosphorylated nsP3 tyrosines were not detected (N.D.) in both untreated or inhibitor treated cellular lysates, potentially due to the lower number of tyrosines when compared to serines/threonines present in nsP3 and/or low sensitivity of the western blot. To demonstrate the importance of nsP3 phosphorylation during viral infection, U-87MG cells were pretreated with the IKKβ inhibitor BAY-11-7082 and infected with VEEV TC-83 (Figure 1C). Treatment with BAY-11-7082 significantly reduced intracellular positive-strand viral RNA levels by ~10-fold at 2 h post-infection (hpi) and >1000-fold at 16 hpi (*p ≤* 0.0001) when compared to untreated cells, consistent with previously published data [31]. These data demonstrate that VEEV nsP3 is potentially phosphorylated on one or more serine and threonine residues when expressed in cells [45,46,47], and inhibition of IKKβ activity diminishes both nsP3 phosphorylation and viral RNA synthesis.

### 3.2. IKKβ Phosphorylates VEEV nsP3 on 13 Putative Amino Acid Residues

**Figure 2:**
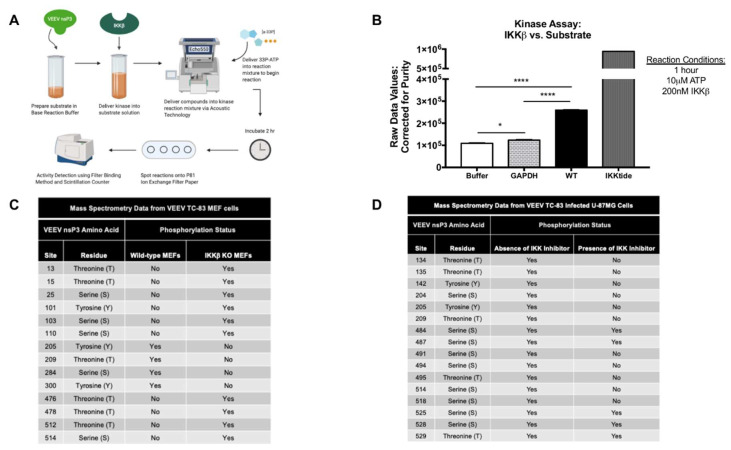
IKKβ activity directly phosphorylates VEEV nsP3. (**A**) Schematic of Kinase HotSpot Assay performed by Reaction Biology Corp. as described in Section 2. Illustration was generated using Biorender. (**B**) VEEV TC-83 nsP3 was expressed and purified from a bacterial expression system as described in Section 2. A cell-free, in vitro assay of IKKβ enzyme vs. purified VEEV nsP3 measured the amount of ^33^P-γ-ATP transferred onto substrate. GAPDH was included as a negative control substrate and ‘IKKtide’, a small validated peptide, was included as a positive control. Graph is representative of the average ^33^P counts measured for duplicate reactions of substrate, corrected for purity, and incubated with 200 nM IKKβ for 1 h (*n* = 2). (**C**) WT or IKKβ^−/−^ MEF cells were infected with TC-83 at MOI of 2 for 1 h and media was replaced after removal of virus. At 6 hpi, total protein lysates were obtained and subjected to LC-MS/MS. Mass spectra were fitted against NCBI reference sequence L01443 for VEEV TC-83 nsP3. (**D**) U-87MG cells were untreated or pre-treated with 1 µM BAY-11-7082 for 2 h, infected with TC-83 at MOI of 1 for 1 h and conditioned media containing 1 µM BAY-11-7082 or standard media was replaced after removal of virus. At 8 hpi, total protein lysates were obtained and subjected to LC-MS/MS. Mass spectra were fitted against NCBI reference sequence NP_740698.1 for putative VEEV nsP3. Phosphorylated amino acid residue sites detected on VEEV nsP3 by LC-MS/MS are listed in panels (**B**,**C**). * *p* < 0.0332 and **** *p* < 0.0001.

Given the large number of predicted phosphorylation sites on nsP3 and the potential for multiple cellular kinases phosphorylating these sites, as well as our previous data demonstrating an interaction between nsP3 and IKKβ [31], we used a kinase activity assay and mass spectrometry to determine whether IKKβ directly phosphorylates nsP3 and identify specific amino acid residues targeted by IKKβ. VEEV nsP3 was purified from an *E. coli* expression system and purity measured at 80%. A cell-free, in vitro enzyme incubation with 200nM IKKβ vs. a single 2 μM dose of nsP3 substrate in the presence of 10 μM ^33^P-ATP was performed for 1 h in duplicate. A schematic of this assay is depicted in Figure 2A.

Controls contained IKKβ alone suspended in reaction buffer to measure any potential IKKβ autophosphorylation signal, and purified GAPDH, a negative control protein not known to be a substrate for IKKβ kinase activity. The positive control measured phosphates transferred on a standard IKKβ substrate peptide (IKKtide) with an input concentration of 20 μM. IKKβ kinase activity was measured by quantitating phosphates transferred onto substrate. The graph in Figure 2B represents raw ^33^P-ATP counts detected by the scintillation counter corrected for purity of input protein substrate. Positive control results indicated 22% phosphorylation of input IKKtide. Our data demonstrate (Figure 2B) phosphorylation of VEEV nsP3 by IKKβ was 110% greater than the levels measured for phosphorylation of GAPDH (*p <* 0.0001) or buffer alone (*p <* 0.0001). The increased phosphorylation signal for VEEV nsP3 in the assay, greater than both the buffer and GAPDH negative control, confirm that the phosphorylation signal in the assay was measured from phosphorylated nsP3 and not from IKKβ autophosphorylation or from nonspecific kinase activity of IKKβ. From our data, we conclude that nsP3 can be directly phosphorylated by IKKβ kinase activity.

Next, we mapped phosphorylation of nsP3 amino acid sites using mass spectrometry. Lysates from TC-83 infected wild-type (WT) and IKKβ^−/−^ (KO) mouse embryonic fibroblasts (MEFs) and lysates from TC-83 infected U-87MG astrocytes untreated or treated with BAY-11-7082, were subjected to tandem LC-MS/MS. Spectra were fitted against NCBI reference sequence L01443 for MEFs and NP_740698.1 for U-87MG cells representing VEEV nsP3 and evaluated for post-translational modification analysis specific for phosphorylation (Figure 2C,D). Four residues (205, 209, 284 and 300) phosphorylated upon TC-83 infection of WT MEFs but did not demonstrate phosphorylation upon infection of IKKβ KO MEFs (Figure 2C), suggesting that these were targeted by IKKβ activity. In addition, ten unique residues were phosphorylated upon IKKβ KO, but not WT MEF infection, which could have occurred due to differential activity of other kinases in this cell line. Eleven residues were phosphorylated upon TC-83 infection of astrocytes, but abrogation of phosphorylation was observed upon BAY-11-7082 treatment (Figure 2D), suggesting that these were targeted by IKKβ activity. Additionally, five residues were phosphorylated upon astrocyte infection regardless of inhibitor treatment, suggesting that phosphorylation at these sites was mediated by other cellular kinases. Other residues in astrocyte cells where phosphorylation was abrogated upon inhibitor treatment were not phosphorylated in WT MEF cells, potentially due to nonspecific, off-target effects of BAY-11-7082 treatment or differential regulation of the activity of various kinases in these cell lines. Collectively, our mass spectrometry data identified thirteen residues that appear to be phosphorylated by IKKβ activity in these cell lines.

### 3.3. Mutation of Residues 204/5, 142, or 134/5 Renders VEEV Deficient in Replication

**Figure 3:**
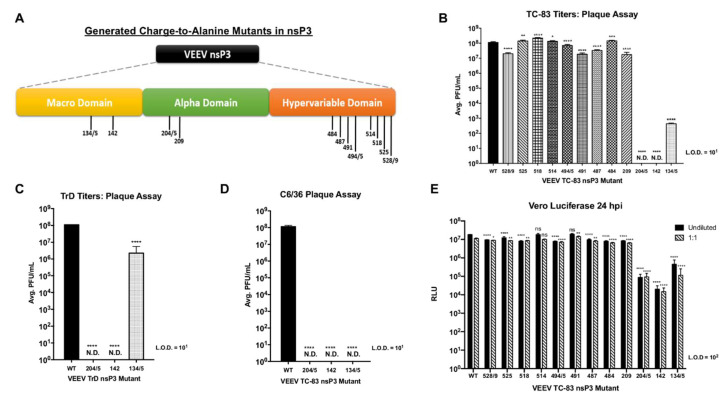
VEEV nsP3 mutants 204/5, 142, and 134/5 are replication-deficient in both TC-83 and TrD strains. (**A**) Graphic illustration of VEEV nsP3 domains. The locations of alanine substitutions for each generated VEEV mutant are represented by their amino acid residue numbers. (**B**) WT and mutant viruses were grown as described in Section 2 and viral titers were measured via plaque assay. ND represents undetectable virus. Mutants 204/5 and 142 were titered in triplicate from four independent experiments (*n* = 12). All other mutants were titered in triplicate from two independent experiments (*n* = 6). (**C**) WT and mutant viruses were grown as described in Section 2 and titers were measured via plaque assay. Graph is representative of one independent experiment (*n* = 3). (**D**) C6/36 mosquito cells were infected in triplicate at MOI of 1 (wild-type and mutant 134/5) or undiluted virus (mutants 204/5 and 142). At 24 hpi, supernatants were collected and plaque assay was performed as described in Section 2. Graph is representative of two independent experiments (*n* = 6). (**E**) Vero cells were infected in triplicate with VEEV TC-83 nsP3 mutants using undiluted virus or diluted 1:1. At 24 hpi, supernatants were collected, and luciferase assay was performed as described in Section 2. Graph is representative of two independent experiments (*n* = 6). * *p* < 0.0332, ** *p* < 0.0021, *** *p* < 0.0002, and **** *p* < 0.0001, ns, not significant.

In order to elucidate the role of individual phosphorylated nsP3 residues in the replication of VEEV, we created TC-83 mutant viruses expressing nano-luciferase [34,35] and containing charge-to-alanine substitutions at 12 phosphorylation sites identified by mass spectrometry. The substitution locations within each nsP3 domain are mapped in Figure 3A. These sites were chosen to encompass residues where phosphorylation was both abrogated and unaffected upon inhibition of IKKβ in WT MEF and astrocyte cells. We did not choose sites phosphorylated exclusively in IKKβ KO MEFs as we did not elucidate other cellular pathways that may be differentially regulated in these cells due to the absence of IKKβ. All subsequent experiments were performed using nano-luciferase-expressing TC-83 wild-type and nsP3 mutant viruses. We measured the growth of viral stocks in order to obtain an infectious titer for subsequent experiments. Surprisingly, following electroporation of equal amounts of in vitro transcribed full-length viral RNA, mutants 204/5 and 142 did not form infectious plaques (Figure 3B) and the production of mutant 134/5 virions was also significantly diminished with a ~10^5^-fold reduction in PF U/mL when compared to wild-type TC-83 (*p <* 0.0001). Other mutants displayed statistically significant reductions in infectious titers when compared to wild-type (*p <* 0.0001); however, the reduction in replication level was modest when compared to that observed for mutants 204/5, 142, and 134/5 (Figure 3B). Furthermore, we generated these mutants in the epizootic wild-type TrD strain of VEEV. Consistent with results obtained using TC-83, mutants 204/5 and 142 did not form infectious plaques (Figure 3C), whereas the production of mutant 134/5 virions was reduced ~50-fold when compared to that of wild-type TrD (*p <* 0.0001). We also assayed these mutants in C6/36 mosquito cells which are competent for IKKβ activity [48,49,50]. TC-83 mutants 204/5, 142, and 134/5 did not form infectious plaques (Figure 3D) when compared to wild-type TC-83 (*p <* 0.0001). Because obtaining an infectious titer via plaque assay as an experimental measure was not possible for mutants 204/5 and 142, luciferase activity was measured 24h following Vero cell infection with VEEV TC-83 nsP3 mutants expressing luciferase, using undiluted viral stocks or 1:1 diluted viral stocks to ensure cells were infected with at least some mutant virions (Figure 3E). Results confirm replication defects for mutants 204/5, 142, and 134/5 with luciferase levels exhibiting a ~100-1000-fold reduction when compared to that of wild-type TC-83 (*p <* 0.0001). However, a positive, luciferase signal, albeit at a low level, suggests that replication at a very low level does occur in cells infected with mutants 204/5 and 142. Taken together, our mutagenesis data suggest residues 204/5 and 142 are vital for VEEV replication in both the attenuated TC-83 and the virulent TrD strain. Residues 134/5 appear to be critical for successful VEEV TC-83 virion production but less significant for virion production in the TrD strain. Additionally, data from mosquito cell infection indicates these residues as critical for VEEV propagation in both mammalian and arthropod host cell types. Because deficient replication phenotypes were confirmed for mutants 204/5 and 142 in the context of the wild-type TrD strain, we used VEEV TC-83 in all subsequent experiments.

### 3.4. Residues 204/5, 142, and 134/5 Are Phosphorylated by IKKβ Activity and Replication-Deficient Mutants at These Sites Are Incapable of Efficient Negative-Sense RNA Synthesis

**Figure 4:**
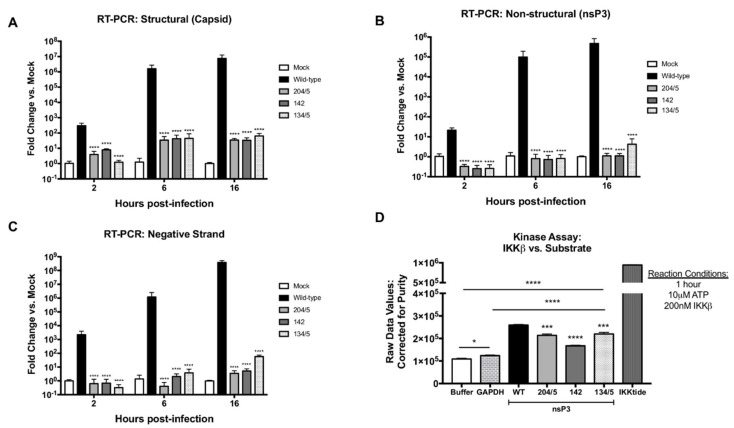
Negative-strand synthesis is curtailed during replication-deficient mutant infection and IKKβ phosphorylates these VEEV nsP3 sites. Vero cells were infected in triplicate at MOI of 1 (wild-type and mutant 134/5) or undiluted virus (mutants 204/5 and 142). At 2, 6 and 16 hpi, RT-PCR to measure positive and negative strand levels was performed as described in Section 2. (**A**) Expression levels of capsid. (**B**) Expression levels of nsP3. (**C**) Expression levels of negative-strand RNA. Graphs are representative of two independent experiments (*n* = 6). (**D**) VEEV TC-83 nsP3 wild-type and mutant proteins were expressed and purified from a bacterial expression system as described in Section 2. A cell-free, in vitro assay of IKK. β enzyme vs. purified VEEV nsP3 measured the amount of ^33^P-γ-ATP transferred onto substrate. GAPDH was included as an inert negative control substrate and ‘IKKtide’, a small validated peptide, was included as a positive control. Graph is representative of the average ^33^P counts measured for duplicate reactions of substrate, corrected for purity, and incubated with 200 nM IKKβ for 1 h (*n* = 2). * *p* < 0.0332, *** *p* < 0.0002, and **** *p* < 0.0001.

We next sought to determine the step or steps in the viral replication cycle at which the mutants 204/5, 142, and 134/5 in VEEV nsP3 negatively affected viral replication. VEEV replication begins after viral entry and uncoating of the genome, following which the positive-sense genomic RNA is translated into the non-structural polyprotein and together, with various host proteins, the replication complex binds to the 3′ conserved sequence element (CSE) on the genomic RNA to initiate synthesis of the negative RNA strand [9,51]. Subsequent cleavages of the non-structural polyprotein into its constituents (nsP1-4) initiate positive-sense RNA synthesis of the full-length genome (49S) and subgenome (26S) via promoters on the negative-strand [51]. Considering the role of nsP3 in the viral life cycle and the low level of luciferase activity observed in Figure 3E, we speculated that receptor binding, entry into the host cell and subsequent disassembly of the nucleocapsid were not compromised by mutations present in nsP3. Thus, we hypothesized that the effect of these mutations in nsP3 manifests during the synthesis of positive- and/or negative-strand RNA. We employed RT-PCR to measure production of positive- and negative-sense strands of the VEEV genome, predicting lower levels of viral RNA synthesis during infection with replication-deficient mutants when compared to wild-type virus infection. Vero cells were infected with wild-type TC-83 or mutant 134/5 at MOI of 1 with undiluted viral stocks used for mutants 204/5 and 142, and levels of positive- and negative-strand RNAs were measured at 2, 6, and 16 hpi. As observed across all time points post-infection, all three mutants were defective in positive-strand RNA synthesis (Figure 4A,B). By 16 hpi, all three mutants displayed a 105-fold reduction in levels of VEEV genome when compared to wild-type (*p* < 0.0001). Because positive-strand genome synthesis of viral RNA was markedly reduced, we questioned whether this was due to a lack of negative-strand synthesis, a critical intermediate step in the VEEV replication cycle. Indeed, all three replication-deficient mutants exhibited significant reductions in levels of negative-strand RNA across all time points post-infection when compared to wild-type (Figure 4C). By 16 hpi, mutants 204/5 and 142 displayed roughly 108-fold reductions and mutant 134/5 displayed a 106-fold reduction in negative-strand viral RNA levels when compared to wild-type (*p* < 0.0001). Further, all three mutants exhibited very modest increases (<5-fold) in both positive- and negative-strand RNA synthesis at later time points post-infection. Taken together, these data demonstrate that mutations at VEEV nsP3 residues 204/5, 142, and 134/5 inhibit negative-strand RNA synthesis.

Given the importance of nsP3 residues 204/5, 142, and 134/5 for successful VEEV replication, we determined whether these sites are targeted by IKKβ kinase activity. To that end, a cell-free, in vitro enzyme activity assay was performed using mutant VEEV nsP3 proteins with alanine substitutions at sites 204/5, 142 and 134/5. Mutant proteins were expressed and purified from an *E. coli* expression system. Purity was measured at 90%, 70%, and 85% for mutant proteins 204/5, 142, and 134/5, respectively. 200nM IKKβ was incubated with 2 μM substrate protein in duplicate as previously described for Figure 2A,B. The graph plots raw ^33^P-ATP counts corrected for purity of input protein substrate (Figure 4D). Our data demonstrate positive signals at least >35% above both buffer and GAPDH counts for all mutant nsP3 proteins (*p <* 0.0001). In comparison to wild-type nsP3, a count reduction of 18% is observed for nsP3 mutation at site 204/5 (*p <* 0.0002), 35% for mutation at 142 (*p <* 0.0001), and 15% for mutation at 134/5 (*p <* 0.0002). From these data, we conclude that nsP3 sites 204/5, 142, and 134/5 can be phosphorylated by IKKβ.

### 3.5. Revertant Viruses Generated Following Serial Passaging of Replication-Deficient Mutants are Cytopathic and Restore Negative-Strand Synthesis

**Figure 5:**
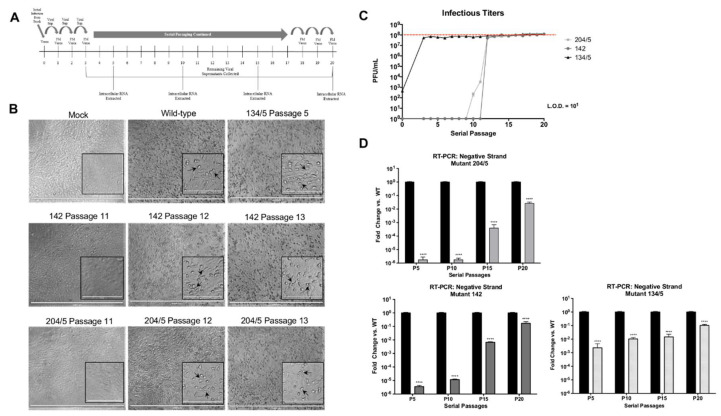
Serial passaging of replication-deficient VEEV nsP3 mutants generates revertant viruses competent for negative-strand synthesis. (**A**) Schematic of serial passaging performed using replication-deficient mutants as described in Section 2. (**B**) Viral supernatants from serial passages 11–13 for mutants 204/5 and 142 and passage 5 for mutant 134/5 were used to infect Vero cells to evaluate cytopathic effect (CPE) as compared to wild-type TC-83 and mock infected cells. (**C**) Viral supernatants from serial passaging were titered in triplicate. (**D**) Negative-strand synthesis levels were measured by RT-PCR as described in Section 2. Graphs are representative of duplicate serial passages prepared in triplicate (*n* = 6). **** *p* < 0.0001.

Thus far, we have demonstrated that VEEV nsP3 sites 204/5, 142 and 134/5 are important for VEEV replication and negative-strand synthesis, and are capable of being phosphorylated by IKKβ activity. To further characterize the importance of phosphorylation at VEEV nsP3 residues 204/5, 142, and 134/5 during replication, we serially passaged these mutants to determine the frequency and nature of potential revertants. Mutants 204/5, 142, and 134/5 were serially passaged on Vero cells at 24 h intervals for a total of 20 passages with supernatants collected daily and intracellular RNA extracted from cells at passages 5, 10, 15 and 20 (Figure 5A). Reversion of mutants to a cytopathic phenotype was observed at passage 12 for mutants 204/5 and 142 and at passage 3 for mutant 134/5 (Figure 5B), and included cellular swelling, blebbing, and rounding causing distorted morphology and lower adherence to culture vessel as compared to mock infected cells.

We next hypothesized that viral replication levels would exhibit plaque and genome copy levels comparable to those observed following wild-type TC-83 infection near passage 12 for mutants 204/5 and 142 and passage 3 for mutant 134/5. Supernatants collected from passage 3-20 were analyzed via plaque assay (Figure 5C). Mutant 142 recovered infectivity within one 24 h passage and regained an infectious plaque titer at passage 11 resulting in 10^8^ PF U/mL by passage 12. Conversely, mutant 204/5 recovered infectivity over three passages as plaque titers rose from un-detectable at passage 9 to 10^8^ PF U/mL at passage 12. Mutant 134/5 recovered infectivity at passage 3 reaching 10^8^ PF U/mL (from 10^2^ PF U/mL initially). Similarly, RT-PCR demonstrated that revertant viruses generated from all three mutants recovered the ability to efficiently synthesize the negative-strand (Figure 5D). Mutant 204/5 displayed a dramatic ~10^6^-fold reduction in negative-strand viral copies when compared to wild-type at passages 5 and 10 (*p <* 0.0001). However, levels rise to ~10-fold reduction by passage 20 (*p <* 0.0001). Mutant 142 displayed a 10^6^-fold reduction in negative-strand viral copies when compared to wild-type at passage 5, but viral RNA levels rise to a less than 10-fold reduction by passage 20 (*p <* 0.0001). Mutant 134/5 displayed a ~1000-fold reduction in negative-strand RNA copies as compared to wild-type at passage 5 but synthesized the negative-strand with less than a 10-fold reduction at passage 20 (*p <* 0.0001). Together, these data demonstrate that mutants 204/5, 142, and 134/5 revert to an infectious phenotype, with both plaque forming units and negative-strand RNA synthesis reaching levels comparable to those observed during wild-type TC-83 viral infection.

### 3.6. Revertant Viruses Restore Wild-Type TC-83 Amino Acid Residues at Positions 204/5, 142, and 135

**Figure 6:**
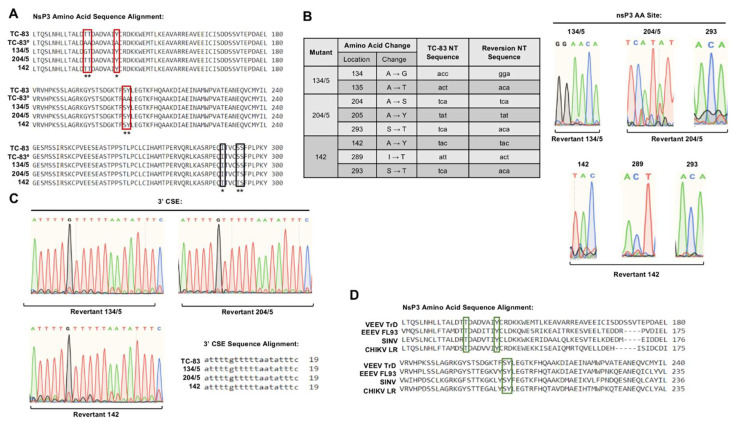
Serial passaging of replication-deficient viruses restores wild-type TC-83 amino acids at positions 204/5, 142, and 135. cDNA specific to nsP3 and 3′UTR was generated, amplified, and sequenced as described in Section 2. (**A**) Protein sequence alignment of nsP3 as compared to wild-type TC-83. ° Indicates the wild-type TC-83 sequence with inclusion of all alanine residues originally substituted in each mutant at sites of interest. * and ** indicate sites of reversion and mutational changes in nsP3. Red boxes highlight original alanine substitution sites and black boxes indicate amino acid changes at additional sites in nsP3. (**B**) Amino acid and nucleotide genomic changes and chromatogram traces observed for each revertant mutant in nsP3. (**C**) Nucleotide sequence alignment and chromatogram traces of CSE for revertant viruses as compared to wild-type TC-83. (**D**) Protein sequence alignment of nsP3 for representative OW and NW alphaviruses. Green boxes highlight conserved amino acid residues of interest.

Because the ability to synthesize negative-strand was recovered upon serial passaging, we speculated that the revertant viruses likely acquired sequence changes in the non-structural protein coding region and/or the 3′ CSE. Therefore, we sequenced nsP3 and the 3′ CSE to determine the mechanistic basis of observed reversions. We hypothesized that the revertants either mutated to phosphorylation-capable amino acids within nsP3 or mutated the 19-nt conserved promoter sequence to compensate for deficient negative-strand synthesis. Intracellular RNA collected at passage 5 for mutant 134/5 and passages 15 and 20 for mutants 204/5 and 142 was sequenced and results were compared to the wild-type TC-83 sequence (Figure 6).

Protein alignment for nsP3 is displayed against VEEV TC-83 with alanine substitution sites highlighted by red boxes (Figure 6A). Surprisingly, mutants 204/5 and 142 exhibited reversion back to the original residues of serine/tyrosine and tyrosine, respectively. Mutant 134/5 reverted to the original threonine at position 135, but position 134 was mutated to glycine rather than the threonine present in TC-83. Interestingly, we identified additional amino acid mutations in nsP3 in revertants 204/5 and 142. Position 293 mutated from serine to threonine in both 204/5 and 142 revertants, while an additional isoleucine to threonine mutation was observed at position 289 in the 142 revertant. Nucleotide analysis of chromatogram traces (Figure 6B) revealed that the genomic sequences of the revertants returned to the original VEEV TC-83 genomic sequences for the rescued alanine substitutions at positions 204/5 and 142. Position 135 reverted to the threonine genomic codon to ACA rather than the TC-83 sequence of ACT. No mutations were detected in the 19-nt CSE’ negative-strand promoter region in any revertant (Figure 6C). Moreover, sequence alignment of Old World and New World alphavirus nsP3 proteins (Figure 6D) shows conservation of phosphorylation-capable residues at positions 135, 204/5, and 142, highlighted by green boxes, which suggests that phosphorylation of these sites may be conserved among alphaviruses. Reversion to phosphorylation-capable residues identical to those found in TC-83 nsP3 suggests that phosphorylation at sites 204/5, 142 and 135 is important for VEEV replication and negative-strand synthesis.

### 3.7. Phosphomimetic Mutations at nsP3 Sites 204/5 or 134/5 Can Rescue VEEV Replication and Negative-Strand Synthesis

**Figure 7:**
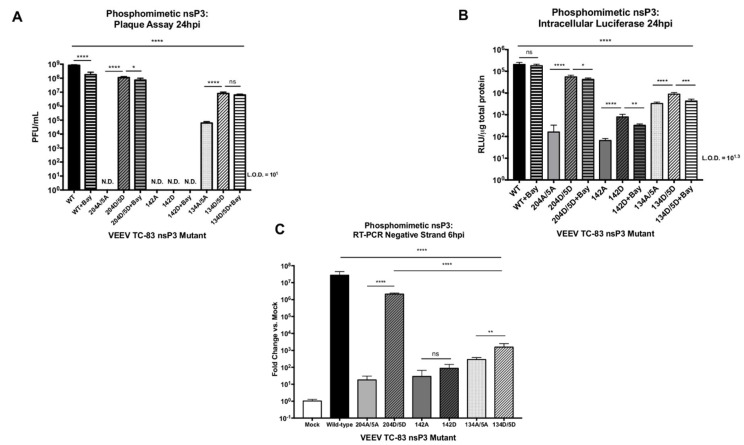
Phosphomimetic mutations at nsP3 sites 204/5 and 134/5 rescue VEEV replication. Vero cells were untreated or pretreated with 10 µM BAY-11-7082 for 2 h, subsequently infected with VEEV TC-83 nsP3 alanine or aspartic acid mutants in triplicate for 1 h, and conditioned media containing BAY-11-7082 or standard media was replaced after removal of virus. Cells were infected at MOI of 1 (wild-type and mutants 204D/5D, 134A/5A, 134D/5D) or undiluted virus (mutants 204A/5A, 142A, 142D). At 24 hpi, supernatants were collected and cellular lysates obtained. (**A**) Plaque assay using viral supernatants and (**B**) intracellular luciferase assay were performed as described in Section 2. ND represents undetectable virus. (**C**) Vero cells were infected with VEEV TC-83 nsP3 alanine or aspartic acid mutants in triplicate at MOI of 1 (wild-type and mutants 204D/5D, 134A/5A, 134D/5D) or undiluted virus (mutants 204A/5A, 142A, 142D). At 6 hpi, RT-PCR to measure negative strand levels was performed in triplicate as described in Section 2. Graphs are representative of two independent experiments (*n* = 6). * *p* < 0.0332, ** *p* < 0.0021, *** *p* < 0.0002, and **** *p* < 0.0001, ns, not significant.

Our serial passage data is indicative of a strong evolutionary pressure to maintain amino acid residues capable of being phosphorylated at nsP3 sites 204/5, 142 and 134/5. However, other factors, notably the identity of the amino acid residue at these sites itself, instead of phosphorylation at these sites, may explain their importance during VEEV replication and negative-strand synthesis. To delineate whether the replication-deficient phenotype observed in Figure 3 and Figure 4 arose from a lack of phosphorylation or the loss of specific amino acid residues, we substituted aspartic acid (D) at nsP3 positions 204/5, 142, and 134/5 to generate phosphomimetic mutants, a technique commonly employed to recapitulate the effect of phosphorylated residues at particular sites [52]. Phosphomimetics were created in a TC-83 nano-luciferase expressing virus backbone and their ability to successfully replicate and synthesize negative-strand RNA was measured. Vero cells were untreated or pretreated with IKKβ inhibitor BAY-11-7082, and infected with mutant viruses at MOI of 1 (wild-type, 204D/5D, 134A/5A, and 134D/5D) or undiluted viral stocks (204/5, 142A, and 142D). At 24 hpi, viral supernatants were collected for plaque assay (Figure 7A), and intracellular lysates obtained for luciferase assay (Figure 7B). Phosphomimetic mutant 204D/5D recovered the ability to generate detectable infectious particles when compared to its alanine substitution counterpart (204A/5A) (*p <* 0.0001), which corresponded with a comparable increase in viral protein production, as measured by luciferase levels. Mutant 204D/5D particle production was only 7.5-fold lower than that of wild-type TC-83 (*p <* 0.0001). Treatment with the IKKβ inhibitor BAY-11-7082 had a minimal impact on the replication of mutant 204D/5D (1.5-fold decrease compared to untreated cells (*p <* 0.0332)). Similarly, treatment with the IKKβ inhibitor minimally reduced viral protein production in mutant 204D/5D infected cells (1.3-fold decrease compared to untreated cells (*p <* 0.0332)). In contrast, IKKβ inhibitor treatment resulted in a 4.6-fold decrease in wild-type replication compared to that in untreated cells (*p <* 0.0001). The phosphomimetic mutant 142D did not produce detectable viral particles (N.D.) (*p <* 0.0001), but did display a 12-fold increase in intracellular luciferase compared to mutant 142A (*p <* 0.0001) (Figure 7B). Treatment with the IKKβ inhibitor reduced luciferase expression in 142D infected cells by 2.4-fold when compared to signal from untreated cells (*p <* 0.0021). The phosphomimetic mutant 134D/5D increased particle production 130-fold PFU/mL (*p <* 0.0001) and luciferase expression 2.7-fold when compared to 134A/5A (*p <* 0.0001). Similar to 204D/5D and 142D, treatment with the IKKβ inhibitor had no significant measurable effect on particle production but did reduce luciferase expression by 2-fold (*p <* 0.0002). Overall, phosphomimetic substitutions with aspartic acid at VEEV nsP3 positions 204/5 and 134/5 rescued infectious particle production which was resistant to IKKβ inhibitor treatment.

We next determined whether negative-strand synthesis was rescued during phosphomimetic mutant replication. Vero cells were infected, and negative-strand RNA levels were measured at 6 hpi (Figure 7C). Phosphomimetic mutant 204D/5D recovered the ability to synthesize the negative-strand RNA by 10^5^-fold compared to 204A/5A (*p <* 0.0001). Further, recovery of RNA synthesis reached levels that were only 13-fold lower when compared to wild-type TC-83 (*p <* 0.0001), and drastically higher than the 10^8^-fold reduction observed with 204A/5A (Figure 4). The phosphomimetic mutant 142D did not significantly recover synthesis of negative-strand RNA, corresponding to the observed lack of detectable virion production in infected cells. Interestingly, despite a significant increase in infectious particle production measured following 134D/5D infection, negative-strand RNA synthesis increased just 5-fold compared to 134A/5A (*p <* 0.0021) and levels remained >10^4^-fold lower than that of wild-type TC-83 (*p <* 0.0001). From these experiments, we conclude that phosphorylation at VEEV nsP3 site 204/5 is essential for negative-strand RNA synthesis, whereas phosphorylation at nsP3 site 134/5 may be important for particle production without playing a major role in negative-strand RNA synthesis.

## 4. Discussion

### 4.1. The Role of IKKβ during the Alphavirus Replication Cycle

The nsP3 of both Old World and New World alphaviruses has been studied due to its capacity for hyperphosphorylation, variability among alphaviruses, and its interaction with a variety of host proteins [9,12]. However, cellular kinases responsible for phosphorylating various sites in nsP3 have yet to be determined [12]. We have previously shown that inhibiting IKKβ activity leads to greatly reduced viral replication following VEEV infection [31]. Here, we extend our previous observations to demonstrate IKKβ-mediated phosphorylation of VEEV nsP3 and elucidate the role of this post-translational modification in the viral replication cycle. First, we demonstrated by western blot that nsP3 phosphorylation levels decrease upon treatment with the IKKβ inhibitor BAY-11-7082. We also used a cell-free, in vitro kinase activity assay containing purified IKKβ and VEEV nsP3 to quantitatively identify nsP3 as a substrate targeted by IKKβ. To our knowledge, this is the first identified cellular kinase responsible for phosphorylating VEEV nsP3. ^33^P-ATP counts for nsP3 were significantly higher than those measured from potential IKKβ autophosphorylation and non-specific IKKβ kinase activity. The lower level of nsP3 phosphorylation vs. the positive control in this assay may be attributed to a lower input concentration of nsP3 substrate vs. the control peptide. Given that the control substrate is a small, highly purified peptide, a greater number of peptide molecules present at a given concentration when compared to the full-length nsP3 protein would also result in a higher observed signal. Furthermore, VEEV nsP3 may not have acquired all post-translational modifications when expressed in a bacterial system. Alternatively, the presence of nsP3 alone, in the absence of other non-structural proteins, may affect the proficiency of its interaction with IKKβ. Similar methodology was employed for SINV nsP3 in which kinase activity was detected following incubation of nsP3 and ^33^P-ATP, with the kinase speculated to be casein kinase II (CKII) [53]. Interestingly, our phosphorylation prediction analysis also identified CKII as potentially phosphorylating two sites, 135 and 204. Using mass spectrometry, we have identified 13 serine, threonine, and tyrosine phosphorylation sites on VEEV nsP3 in two distinct cell types that appear to be phosphorylated by IKKβ. Evaluation of mutant nsP3 proteins, 204/5, 142, and 134/5, by a cell-free, in vitro kinase activity assay confirmed that these sites can be phosphorylated by IKKβ kinase activity. Additionally, nsP3 residues that are phosphorylated regardless of the presence or absence of the IKKβ inhibitor provide evidence that nsP3 can be phosphorylated by the activity of at least two host kinases. Indeed, our phosphorylation prediction analysis identified a variety of kinases potentially phosphorylating nsP3, including at the same residues, for example, CKII and IKKβ at sites 135 and 204. Given that VEEV replication is not completely abrogated upon BAY-11-7082 treatment or in IKKβ^−/−^ cells [31], multiple cellular kinases probably phosphorylate nsP3 in the absence of IKKβ activity. Thus, our data identifies IKKβ as one kinase phosphorylating VEEV nsP3 at multiple sites but does not demonstrate that this interaction is absolutely essential for VEEV replication.

Our conclusions regarding the role of nsP3 phosphorylation during VEEV replication are supported by those obtained from a previous study using VEEV, in which 53 potential phosphorylation sites in the nsP3 HVD were mutated. That study revealed minimal effects on viral replication and concluded that the phosphorylation state of this domain is inconsequential for replication [14]. Similarly, the mutation of eight phosphorylation sites in nsP3 HVD in our study also demonstrated minimal effects on viral replication. However, our study extends the analysis of nsP3 phosphorylation to the macrodomain and the AUD of nsP3 and identifies phosphorylation sites in both these domains that are critical for VEEV replication.

### 4.2. NsP3 Residues 204/5, 142, and 135 Are Critical for Negative-Strand Synthesis

Our research identified three phosphorylation sites in VEEV nsP3 at amino acid positions 204/5, 142, and 134/5 at which charge-to-alanine mutations dramatically diminished viral replication when introduced into the TC-83 or TrD strains of VEEV. Furthermore, the abrogation of phosphorylation-capable residues at any one of these sites reduced negative-strand synthesis during VEEV TC-83 infection, suggesting that nsP3 activity during replication could require multiple phosphorylation events. However, the collective impact of these residues may be cell-type dependent and phosphorylation at these residues may also be temporally regulated. For example, sites 142 and 134/5 were phosphorylated in astrocyte cells at 8 hpi but absent in the wild-type MEF cells at 6 hpi. This phenomenon could suggest that differential phosphorylation at certain nsP3 residues may occur at different time points post-infection in different cell types during replication. Furthermore, all three mutants were incapable of virion production following infection of mosquito cells, suggesting that these sites are required for viral growth in multiple cell types.

Our data supports research performed in a heat-resistant (HR) strain of SINV where a mutation at position 268 from alanine to valine in the N-terminal conserved region of nsP3 rendered the virus defective in negative-strand synthesis. Furthermore, double recombinant genomes allowed reactivation, suggesting that the impairment of negative-strand synthesis was due to prevention of active replication complex formation early during replication [41]. Similarly, a glycine substitution at site 68 resulted in blockage of overall SINV nsP3 phosphorylation and was correlated with diminished negative-strand synthesis [54]. Furthermore, two deletions in Semliki Forest virus nsP3 at positions 344 and 345 in the HVD, individually and doubly deleted, resulted in a considerably diminished rate of viral RNA synthesis, albeit with successful virion production [15]. One potential mechanism by which nsP3 phosphorylation may support viral replication is by enabling interactions with host factors that may be involved in the viral replication cycle. For instance, Fragile X syndrome family proteins interact with VEEV nsP3 whereas Chikungunya virus (CHIKV) and SINV nsP3 exploit the G3BP family of proteins to enable formation of replication complexes [17]. Mutation of residues 260 and 261 in CHIKV nsP3 can abrogate viral replication, and in silico modeling suggests these sites may play a role in nsP3 structural integrity and/or interaction with host cellular components [55]. More recently, the HVD of CHIKV nsP3 was shown to contain two SH3 domain-binding motifs and point mutations in these sites not only reduced viral replication but also eliminated interaction with SH3 domain-containing host proteins CD2AP, BIN1, and SH3KBP1 [56]. Binding of host proteins with the SH3 domains was found to cause allosteric effects in the HVD of CHIKV nsP3, which affected the interaction with G3BP family proteins [56]. Our data parallels the mechanistic results described in these studies where mutations in nsP3 that abrogate phosphorylation result in decreased negative-strand RNA synthesis which may occur due to abrogation of interactions between nsP3 and host proteins facilitated by nsP3 phosphorylation.

### 4.3. The Evolutionary Importance of Phosphorylation Competence at Positions 204/5, 142, and 134/5

To further highlight the importance of replication-deficient nsP3 sites 204/5, 142, and 134/5 for successful viral replication, we serially passaged these mutants to generate revertants that restored infectivity and negative-strand synthesis. Interestingly, all three revertants restored the alanine mutations back to the original phosphorylation-capable residues present in wild-type TC-83 at these sites, which suggests that viral fitness requires the presence of phosphorylation competent amino acids at these sites. However, mutant 134/5 did not mutate amino acid site 134 back to the original threonine and instead mutated to glycine, indicating that of the pair, amino acid site 135 is probably more important for successful VEEV replication. Furthermore, our phosphorylation prediction analysis identified only site 135 as a residue targeted for phosphorylation. Intriguingly, alignment of SINV, CHIKV, and eastern equine encephalitis virus (EEEV) nsP3 sequences with VEEV nsP3 shows conservation of residues at 204/5, 142, and 135 among these alphaviruses, potentially suggesting that phosphorylation at these residues represents an evolutionarily conserved mechanism for successful replication.

Additionally, data from phosphomimetic mutants suggests differential roles for phosphorylation at individual sites in the replication cycle. Phosphomimetic mutant 204D/5D restored infectious particle production and this correlated with a restoration of negative-strand RNA synthesis indicating that phosphorylation at positions 204/5 in VEEV nsP3 is more important for negative-strand RNA synthesis than the identity of the amino acid residues. Indeed, as aspartic acid substitution at this site mimics a constantly phosphorylated residue, this suggests that constant phosphorylation of site 204/5 is potentially required for negative-strand synthesis. Additionally, treatment of 204D/5D with the IKKβ inhibitor BAY-11-7082 displayed a minor reduction in infectious particle production further suggesting that phosphorylation at this site is important for successful replication and negative strand synthesis. The lack of inhibition observed following treatment of 204D/5D infected cells with BAY-11-7082 may reflect phosphorylation of nsP3 by multiple kinases that presumably overcome the inhibitory effect of BAY-11-7082 on IKKβ activity.

In contrast to the rescue of replication observed with mutant 204D/5D, the phosphomimetic mutant 142D failed to restore infectious particle production or negative-strand RNA synthesis rendering interpretation of the role of phosphorylation at this site inconclusive. A mimic at this site can only represent a constitutively phosphorylated residue. Given this, we can only conclude that alanine substitution at this site abrogates viral replication and negative strand synthesis, that the aspartic acid substitution may have destabilized nsP3, that a residue mimicking constant phosphorylation at nsP3 site 142 does not appear to restore viral replication, and/or that the use of a phosphomimetic at this site cannot duplicate the natural function of a phosphorylated residue at this site in nsP3.

Most interestingly, phosphomimetic mutant 134D/5D infection resulted in a significant rescue of infectious particle production, but not negative-strand RNA synthesis. Treatment of 134D/5D with BAY-11-7082 demonstrated no significant reduction in infectious particle production supporting our previous conclusion that phosphorylation at this site is important for viral replication. Taken together with the alanine substitution data where detectable infectious particle production but negative-strand RNA levels comparable to mutants 204/5 and 142 are observed, this suggests that phosphorylation at nsP3 site 134/5 is important for infectious particle production but the identity of the amino acid residue at this site may be important for negative-strand synthesis. Alternatively, a threshold effect may apply such that the small observed increase in negative-strand level above a given amount during 134D/5D infection may be sufficient to increase infectious particle production. Another possibility, given the large observed increase in PFU production with a correspondingly small change in negative strand levels, is that phosphorylation at nsP3 site 134/5 results in an increase in the efficiency of viral genome packaging into virions. In a study using capsid protein mutants that altered its ability to package viral genomes, compensatory mutations in non-structural protein 2 (nsP2) were observed that corresponded with increased viral titers [57]. Results suggested that nsP2 may be involved in RNA encapsidation or could mediate presentation of viral genomes for packaging. Therefore, phosphorylation of nsP3 at site 134/5 could mediate nsP3 functions different from its role in viral RNA synthesis.

Lastly, defining the mechanism by which negative-strand synthesis is curtailed may provide insights into the initiation and regulation of this activity during infection. It is unknown as to whether these mutations affect replication complex formation, binding to the viral genome at the positive-strand 3′ CSE promoter, or curtail the rate of initiation or elongation of negative-strand synthesis, and whether they do so by perturbing nsP3 function or abrogating interactions with host factors involved in negative-strand synthesis. Further, determining the means by which an increase in infectious particle production can occur without a significant increase in negative-strand RNA levels could describe a role for nsP3 outside of the viral replication complex. Future experiments using these mutants will be performed to further elucidate the molecular mechanisms by which nsP3 may regulate negative-strand synthesis and infectious particle production.

## Figures and Tables

**Table 1:** Positive- and Negative-strand primers used for RT-PCR. Primer/probe sequences specific to VEEV positive-strand and negative-strand RNA developed using IDT PrimerQuest tool and utilized for RT-PCR analysis.

Target	Nucleotide Region of Interest	Type	Sequence (5′ → 3′)	Start Position	Length (Bases)	Amplicon Size (bp)
**Capsid**	7562–8386	Forward Primer	TCTGACAAGACGTTCCCAATCA	7931	22	55
		Reverse Primer	GAATAACTTCCCTCCGACCACA	7985	22	
		Probe	TGTTGGAAGGAAGATAAACGGCTACGC	7951	25	
**Non-structural protein 3 (nsP3)**	4032–5702	Forward Primer	CCATATACTGCAGGGACAAGAA	4450	22	101
		Reverse Primer	CACTGAAGAGTCGTCGGATATG	4550	22	
		Probe	ATGACTCTCAAGGAAGCAGTGGCT	4479	24	
**Negative-Strand**	N/A	Reverse Transcription: T7-F-Neg	GCGTAATACGACTCACTATATCCGTCAGCTCTCTCGCAGGTA	N/A	42	N/A
		Forward Primer: T7	GCGTAATACGACTCACTATA		20	
		Reverse Primer: R-Pos	ACAGGTACTAGGTTTATGCG		20	

Not applicable (N/A) for region of interest.

**Table 2:** Primers used for cDNA generation and sequencing of serially passaged mutants. Primer sequences utilized for cDNA generation, PCR amplification, and sequencing of VEEV RNA extracted from revertants during serial passaging.

Genome Segment	Step	Type	Sequence (5′ → 3′)
**Non-structural protein 3 (nsP3)**	RT	T7-TC83-nsP3-R Pos	GCGTAATACGACTCACTATATGCACCCGCATCAAACCGTC
	PCR	Forward Primer: TC83-nsP3-F	GCACCCTCATATCATGTGGTG
		Reverse Primer: T7	GCGTAATACGACTCACTATA
	Sequencing	Target basepair: 4032	GCACCCTCATATCATGTGGTG
		Target basepair: 4830	TGCTTGTGCATCCATGCCAT
		Target basepair: 5630	AAATAGGGTGATTACAAGAG
**CSE on 3′ end of VEEV Positive Strand**	RT	T7-TC83-CSE-R Pos	GCGTAATACGACTCACTATAGAAATATTAAAAACAAAATCCG
	PCR	Forward Primer: TC83-CSE-Pos-F	GGATCAGCCGTAATTATTATAATTGGC
		Reverse Primer: T7	GCGTAATACGACTCACTATA
	Sequencing	Target basepair: 11246	GGATCAGCCGTAATTATTATAATTGGC
